# “I Wish I Had Help Earlier. We Could Have Been Happier Sooner.” Overcoming the Bystander Effect in the Care for Alcohol-Dependent Parents

**DOI:** 10.3389/fpsyg.2021.656320

**Published:** 2021-07-02

**Authors:** Anke Snoek, Boukje A. G. Dijkstra, Wiebren Markus, Margreet Van der Meer, Guido De Wert, Dorothee Horstkötter

**Affiliations:** ^1^Department of Health, Ethics and Society, Maastricht University, Maastricht, Netherlands; ^2^Nijmegen Institute for Scientist-Practitioners in Addiction (NISPA), Nijmegen, Netherlands; ^3^IrisZorg (Netherlands), Arnhem, Netherlands; ^4^Tactus Verslavingszorg, Deventer, Netherlands; ^5^School for Mental Health and Neuroscience and Department of Health, Ethics and Society, Maastricht University, Maastricht, Netherlands

**Keywords:** alcohol misuse, parenting, child well-being, qualitative research, bystander effect

## Abstract

Parental alcohol dependency is associated with risks for the well-being of their children. However, guiding these families to support is often complicated. We interviewed 10 alcohol dependent parents, and held 3 focus group interviews with child welfare social workers, and alcohol and other drug workers. We identified a reluctance to act among professional and non-professional bystanders. Family members, neighbours, teachers, and general practitioners are often aware of parental drinking problems, but are reluctant to discuss them with the parents or to alert services designed to support families. The aim of this paper is to share the experiences of parents and show that parents appreciate interventions if done in a certain manner. Although parents were reluctant to discuss their drinking problem, they considered these problems as symptoms of underlying severe distress. They were highly motivated to get help for these underlying problems and wondered why they were not questioned about their distress by those around them. The silence of others reinforced pre-existing feelings of worthlessness and hopelessness. In this paper we analyse other's hesitation to intervene as a form of the bystander effect, and make suggestions on how this bystander effect can be overcome.

## Introduction

Substance misuse can have severe detrimental effects on the life of users themselves. When they are a parent, substance misuse can also make it difficult to meet all the responsibilities of parenthood and can negatively affect their children's lives. Children of substance dependent parents often report a lower well-being later in life compared to children of non-substance dependent parents (Barnard and Barlow, 2003; Kroll, 2004).

Although these families seem in need of professional support, the care for these families seems to be complicated for several reasons, for example because of taboos surrounding parental addiction (Young et al., 2007; Osterling and Austin, 2008; Forrester and Harwin, 2011; Oliveros and Kaufman, 2011; Niccols et al., 2012).

We did an explorative study in the Netherlands to investigate the views of parents and professionals on the main bottle necks in care, and how they imagined possible solutions. In this paper, we want to discuss one of the findings of our study: reluctance to act, which was found both among professional and non-professional bystanders, that is people close to struggling families like neighbours, befriended parents at school, friends, family.

We will first outline our method. Second, we will look into the frustrations of professionals and parents regarding intervention, and their observations of the reluctance of various kinds of bystanders to acknowledge apparent signs of distress. Combining insights from studies on the bystander effect with the needs and hopes of parents, we will make recommendations on how the bystander effect can be overcome. We will show how bystanders can play an important role in motivating families to seek support by showing compassion and acknowledging the broader life struggles of the parents.

## Method

The aim of the broader research was an explorative study in how the care for families with parental alcohol dependency could be improved. The aim of this paper is to look into the findings on reluctance to act, and the potential positive role bystanders can play.

We conducted a series of qualitative interviews in the Netherlands with 10 alcohol dependent parents aged 30–50, with a mean age of 42 years. Three parents were recruited in a long-term residential family treatment program that is specifically for parents struggling with addiction. Seven parents were recruited in a short-stay (from 2 weeks up to 3 months) detoxification facility. These parents underwent detoxification followed by in-patient therapy. We interviewed six alcohol dependent mothers and four fathers. At the time of the interviews, parents had between one and four children, aged 1–22 years. Six parents had an alcohol dependency only, four parents currently had an alcohol dependency only but used to be addicted to other substances previously (heroin, cocaine, GHB, speed, diazepam).

Interviews were conducted individually at their homes or within the care facility, depending on the respondent's preference. Interviews lasted about 1 h. We used a combination of a semi-structured questionnaire and a timeline narrative approach (Berends, 2011). Respondents were first invited to tell their life storeys and to elaborate on life events relevant to their substance dependency and parenthood. Additionally, specific questions were asked about their experiences with the treatment facilities and child welfare. They were also asked about strategies that would, according to them, be necessary to improve care. We decided to focus specifically on alcohol dependency, because this is the most prevalent type of substance misuse for parents, and studies have indicated that alcohol misuse is under-detected and its impact is underestimated (Harwin and Forrester, 2002, p. 41).

We also conducted three focus groups with professionals: two with child welfare workers (*n* = 5), and one with workers in alcohol and other drug (AOD) services (*n* = 6). Professionals were asked to reflect on how care could be improved for families in which there is parental alcohol abuse.

All interviews were recorded, fully transcribed verbatim, and analysed in NVivo, a software package that facilitates analysis of qualitative data. Selected quotes were translated from Dutch to English by the first author.

The qualitative data is analysed at four levels: (i) interpretative content analysis, to identify typologies of ethical issues, (ii) thematic analysis, built on open and axial coding, to identify factors across different typologies; (iii) values-discourse analysis, to identify ethical frameworks and thinking strategies; and (iv) a narrative analysis of case studies (Charmaz, 2011; Wertz et al., 2011). Two researchers (AS & DH) analysed the data independently, to insure inter-rater reliability. Inter-rater reliability was established in a dialogical rather than a quantitative way. This paper mainly uses insights from (ii) thematic analysis and (iv) narrative analysis.

### Early Intervention, Presumed Care Avoidance, and the Knowledge of Bystanders

The first prominent finding was that professionals identified the lack of early intervention as the main barrier to effective care. They presumed that this difficulty to early intervention was caused by parental care avoidance: parents concealed their problems from professionals until their situation had severely escalated. However, parents did not support this view of being care-avoidant. Secondly, we found that also parents expressed the wish that they had had help earlier. This seemed a promising finding: that both parents and professionals hoped that these families found their way to care sooner. But how can this be realised? The third remarkable finding was that both parents and professionals described that those close to families with parental substance dependency were often aware of the struggles of these families, but did not know what to do. In the rest of this paper we will explore the significance of this group of bystanders in the process of motivating alcohol dependent parents to seek care. We will first discuss the observation of professionals and parents that bystanders often are aware of the distress these families face, and could potentially play an important role in motivating parents to seek treatment. We will then look at the lessons learned from research on the bystander effect, and make recommendation for policies based on promising best practises.

### The Knowledge of Bystanders

What puzzled both parents and professionals alike was that, in their experience, those close to struggling families were often aware of parental distress but were reluctant to act on these signals. This held true for professionals such as General Practitioners (GPs), teachers at kindergartens and schools, health care providers, as well as non-professionals like family members, neighbours, friends, and befriended parents at playgrounds and school. Professionals and parents stated that they thought that these bystanders could play an important role in helping parents to seek help, professionally and non-professionally.

One of the child welfare workers described a case in which a mother struggled with borderline personality disorder and a severe drinking problem. At a certain point, her children, who were 8 and 12 at that moment, became so scared of her that they locked themselves in their room. The mother literally tried to smoke them out by creating a fire before their bedroom door. It was only at this crisis point that the child welfare services got involved.

How bad have things become if the mother needs to almost literally burn the house down before we help the children. That is outrageous. You asked about ethical dilemmas… Then I wonder… How can this happen? While the network knows about it, the GP knows about it. The school has suspicions. Yes, then I think that our system of care in the Netherlands is severely negligent. (Timothy, child welfare worker)

The case ended with the children being placed out of the house to live with their uncle, and the mother drinking herself to death. Another child welfare worker described a case that made a strong impression on him. He was intensively working with a mother of a 2 year old child. He knew she had mental health problems, but she successfully managed, with the help of her father, to conceal her drinking problem. He found out by accident when he paid an unannounced visit. It shocked him that he failed to notice it, and that others—the cleaning lady, childcare, the father—knew but did not notify him.

Professionals themselves also described that they sometimes found it hard to intervene, or to discuss parenthood.

When in my personal life, I hear storeys about a mother who drinks too much, I also think ‘do I have to report that? I don't even know that person.’ (…) I understand how hard it is. As child welfare agents, we ask a lot from people. Even for me, as a professional, I sometimes find it hard, there are no easy answers. (Samantha, child welfare professional)

I remember well that at a certain moment I had to discuss with a client something that was not functional in their relationship with their children. (…) The moment you start talking about that, you are going to talk about the most vulnerable part of a client. You're going to talk about being a parent and their relationship with their kids. I had to cross eight barriers before I finally had the guts to talk about it with. (Oscar, addiction professional)

Several parents stated that they would pick up their children from school or walk around the neighbourhood while being intoxicated, hence no one ever started a conversation about it with them.

I would wake up in the morning and feel miserable. I would drink two glasses of liquor. After that, I would make a cup of tea, but after the tea, liquor again. I would drink like that all day. Preferably not until I was smashed, but I couldn't always control it. Looking back, I think I was more intoxicated than I thought. (…) But I don't know how bad it was, because no one ever said something. (Barbara)

Drinking apparently changed me, because people in the neighbourhood started to eye me, but no one ever said something. (Steve)

Another respondent, Monique, is a 36 year old single mother with three children aged between 8 and 13. She drank to cope with severe domestic violence. She states:

I wish I had help earlier. We could have been happier sooner. (…) But I wasn't capable myself of seeking help. But think about it: the school knew I was drinking. The police had been at our door so many times, but they never did anything to help the children. (…) I wish they had intervened earlier. (Monique)

She felt that it was clear that there were enough signals that she was struggling but no one around her reached out to help her:

Look, if the police got 23 reports of domestic violence, why didn't they start enquiring after the third time? Why didn't they think: ‘Let's have a look, let's talk with this lady.’ (…) Even if from the outside the children look alright. (…) If the parent smells like alcohol in the morning, the school should report it sooner. (Monique)

One of Monique's neighbours later told her that already for 5 years she had suspicions that Monique was struggling.

The experiences of support workers and parents showed that those surrounding these families often were aware of the problems, but were not sure what to do or how to start a conversation. Studies from child abuse seem to support these findings (Paquin and Ford, 1996).

### Lessons Learned From the Bystander Effect

Very little research has been done on the deliberations and experiences of people surrounding alcohol dependent parents. Until more research is done, we will draw parallels with research on the bystander effect, and with research on why professionals are reluctant to comply with mandatory reporting laws.

The bystander effect was first described by Latane and Darley (1968) when a group of bystanders failed to intervene when a young woman was killed in the middle of the street. Over 30 people reportedly witnessed the event, but no one helped.

When analysing the interviews, we noticed that the situations that the respondents described seemed to be a variation of the bystander effect: many people in their environment noticed their distress and alcohol abuse, yet no one talked to them about this and no one intervened. The situation the parents describe differs from a paradigmatic case of the bystander effect in two ways. First, the bystander effect only describes how bystanders fail to protect someone from a perpetrator. In the case of parental alcohol dependency we are not merely interested in whether or not bystanders should intervene when parents harm their children; rather, we are mainly interested in the period before that: responding to parental distress in itself to prevent (potential) future harm to children, without regarding the parent as a perpetrator but as someone in need of care.

Second, the bystander effect describes how strangers fail to intervene, whereas we are concerned with people close to the families who fail to intervene. Some of the literature on the bystander effect in reporting child abuse is useful here, because this literature reflects on the complex nature of people close to a family intervening in family matters (Christy and Voigt, 1994; Hoefnagels and Zwikker, 2001; Taylor et al., 2016). However, our main point of interest is different and we focus on the distress that precedes or facilitates any alcohol abuse.

Now that we have identified a variation of the bystander effect surrounding parental alcohol dependency and any underlying distress, the question arises how the effect can be overcome. Latane and Darley (1968) analysed why bystanders fail to intervene, and itemised five steps in successful intervening. First, the bystander must notice that something is happening. Second, they have to interpret the event as a matter of urgency. Third, the person must decide that it is his or her responsibility to intervene. Fourth, the bystander has to decide how he or she wants to intervene. Finally, the intervention must be carried out.

Research by Christy and Voigt (1994) on how to overcome the bystander effect in reporting child abuse revealed that feeling responsible and being certain about how to intervene predicted whether bystanders would intervene. Our interviews extend these findings to the case of parental distress. Based on our interviews with parents, we will outline that, and how, parents would like others to intervene.

Our goal is not to shame loved ones of people struggling with alcohol dependency. We also do not wish to diverge the responsibility to seek help from parents to others. Rather we try to show that parents struggling with alcohol dependency often long for someone to support them, rather than actively avoid care, and we will provide strategies of how people could best guide them to care. The impression we have is that loved ones and bystanders often want to help, but do not know how and that parents indeed are very reluctant to some kinds of interventions and ways of being approached but are very open if not even desperate for other approaches.

### Bystanders: Respecting Privacy or Reinforcing Feelings of Worthlessness?

Before we outline how parents would like bystanders to intervene, we will first contemplate on the reasons bystanders might have not to intervene. We do not know why bystanders do not intervene. We know from the literature that in case professionals fail to comply to laws of mandatory reporting, they do so because they are afraid that they unjustly interfere in private matters, and that this will harm the care relationship (Kalichman, 1999; Alvarez et al., 2004) more than it will contribute to a bettering of the overall situations of those they might report about. Something similar might hold true for non-professional bystanders.

There is a certain reluctance to intervene in private matters, and problematic consumption of alcohol and domestic violence are often (erroneously) perceived as private affairs. One way to respond to these signs of distress is by looking the other way andgive people some privacy regarding their family problems. In general, people wish to respect other's privacy and autonomy (Kalichman, 1999; Alvarez et al., 2004). However, the parents we interviewed experienced this concern for privacy not as signs of respect and dignity, but instead rather as forms of apathy and indifference. Unfortunately, by turning a blind eye to the problems experienced by the families, the parents' pre-existing feelings of worthlessness were exacerbated.

One of the parents we interviewed, explained that the very fact that no one offered help even though many people knew about her struggles—first with her violent partner, later with her being traumatised and isolated, and finally about her abuse of alcohol—actually reinforced her feelings of worthlessness and hopelessness. She acquired this low self-esteem already as a child, but being ignored in her struggles reinforced her negative feelings about herself and made her feel unworthy of help. She felt trapped in a vicious circle.

The only message I got during my childhood was: ‘you shouldn't have existed. You are worthless and will never amount to anything’. If that is the only experience you have ever had in your life then the fact that others do not seem to respond to your struggles only reinforces this feeling. You think at school: ‘Look, they never ask how I am doing, they just let me be’. (Monique)

For Monique, recognition of her underlying problems and an offer of help would have been key to addressing her drinking problem earlier. These findings are in line with earlier observations made by Kalichman (1999) who argues that if clients know that professionals could help or could report problems, but the professionals refrained from doing so, the clients might interpret this as a sign that the professional is unwilling to get involved, or as a sign that the clients might be unworthy to receive care and help. Something similar seems to hold true for non-professional bystanders.

The interviews with parents seemed to indicate that parents do appreciate intervention. However, there seems to be a certain tension between the statements of the parents that they wished other intervened, and the general image of these parents as care avoidings concealing their problems. We asked parents what they thought of this tension, and they indicated that they could feel very threatened by certain interventions. However, they also indicated how they would like to be approached.

### Breaking the Bystander Effect: Compassion for Life Distress

Parents indicated that they felt very threatened by interventions directly questioning their drinking or their parental skills.

When she was asked to specify who should have intervened, and how, Monique acknowledged that initially she would not have been open to receive help for herself or for her children:

I knew I would have become very angry. That was also what the head of school said to me. She said: ‘I knew you were drinking, and I knew you had problems, but the kids seemed to be doing okay. (…) If I would have called Child Welfare Service, you would have gotten very angry, and maybe you would have physically attacked me’. And yes, I think I would have done that, because if I had been drinking, I would have been furious [with her intervention]. Don't threaten my children, or you will get into a fight with me. And that is the ambivalent part: on the one hand, you are hoping so hard for someone who pressures you to seek help.

Monique had clear ideas on how parents should be approached in order to make it easier for them to accept care. She stated that conversations from concerned bystanders should focus on care and be done with compassion; accusations or criticism should be avoided. Offers of support should not primarily focus on her drinking, but on the underlying problems.

They should have a conversation with the parent. Not in an accusing way, like ‘oh, you are an alcoholic’ (…) but: ‘Is there something we can do to help you?’ (…) if the intention is care, and not accusation, that is very important. (…) When you are addicted, you feel immediately criticised as a parent ‘ah, you are not taking good care of your children’, I am very sensitive to that.

Offering support for her personal problems would have been a good way to also address her alcohol abuse and its effect on her family life.

Respondent Steve had a similar experience. When asked if anyone had ever offered him help with his drinking problem, he said “no.” When questioned whether or not he would have found it helpful or not, he replied:

I wouldn't have liked it, but I think it would have helped me. (…) If my brothers and my sister, if they would have said: ‘Steve, what is happening with you? Is there something that is worrying you? Are you bottling things up?’ Then maybe I could have shared my feelings surrounding my father's death, and things might have been different. Then I would have had support earlier. (…) Then I wouldn't have decided to go overseas [to smuggle drugs]. Then I wouldn't have had the gaol sentence. I could have had a more normal life than now.

He reported that his son's non-judgmental attitude had motivated him to seek care. His son told him:

Dad, I don't mind if you start drinking again. It doesn't matter, I will still visit you, because I know who you are, and you always have a good attitude. I will love you just as much, only you will not be able to experience much of my life. Since if you keep drinking, you will only have three years left to live.

The respondents described their drinking problems as a response to severe distress in their lives. For example due to persistent domestic violence, or childhood trauma, but often due to complicated, long-term life adversities. One parent described how years of fertility treatments, combined with complications during childbirth and severe behavioural problems of her daughter, made her quit her job, put strains on her marriage and resulted in her drinking habits. Another respondent described how his first child suffered from reflux and would scream for hours and days on end, triggering a war trauma he thought he had dealt with 10 years ago. Because he no longer worked in the army, he was not sure if he could confine to a therapist about his trauma, or whether he was legally obliged to keep silent about this experiences. This, in combination with some deaths in the family, and the affair of his wife fuelled his drinking habits. Drinking was a coping strategy for most parents that helped them to deal with their issues in the short-term, but obviously in the long term worked seriously against them. Although they knew that their drinking habits were unhelpful, they would prefer others to address their underlying distress rather than their drinking problems as such.

Our interviews showed that parents would have liked bystanders to respond to their distress. Parents stated that they did not mind if others interfered with their private matters, as long as their intention was to support and not to judge, and as long as their questions were aimed at teasing out the underlying stresses and not solely on their drinking habits. This attitude of genuine interest in the distress parents experience could have motivated them to seek treatment for their trauma and as a consequence possibly also for their drinking habits at a much earlier phase.

### Inspiring Practises

As Christy and Voigt (1994) argue, the spell of the bystander effect can be broken by increasing a sense of responsibility and educating people on how to act. Parents indicated that they are in need of compassionate response by bystanders. There are several practises in public health that already do focus on these aspects and that might inspire also good practise in the context of guidance to care of alcohol dependent parents. The Australian campaign “R U OK?” (Mok et al., 2016; Ross and Bassilios, 2019) which encourages people to ask those in their community who might be distressed: “Are you okay?” could be a relevant example in this regard Even though the goal of that campaign is quite different and targets suicide-prevention, the means invoked to that aim seem to be inspiring for the current context as well. The focus is on giving bystanders some—mental—resource to reach out and thereby to open up a way for distressed people to feel connected. In a similar way, also the T‘Mental Health First Aid' (MHFA) program is an early prevention program that aims at making the general public feel more comfortable to approach people who apparently are in distress and to identify signs and symptoms of distress (Kitchener and Jorm, 2006; Hadlaczky et al., 2014).

Most of the current public health campaigns around distressed families focus on the importance of reporting. Again, no data are available on why laypeople fail to report, but there are some data on why professionals feel uncomfortable with mandatory reporting systems, although in many countries they are obliged by law to do so (Thompson-Cooper et al., 1993; Goldsberry, 2001; Lecluijze, 2015). Professionals report that they often are not sure whether the signs they see are grave enough to report, they fear that they unjustly accuse someone, they are unsure what will happen with their report and they fear that the situation would worsen for the already struggling family. In sum, they fear that if they officially reported the parents they would harm their caring relationship with the family and in the end to more harm than good (Kalichman, 1999; Alvarez et al., 2004). People often feel uncomfortable with reporting, but do not know what they could do otherwise. As a consequence, many fail to intervene or provide support altogether. Our study shows how concerned bystanders can engage with parents, that way, reporting might probably become less pressing as more parents become motivated to seek support. Otherwise, bystanders could discuss with parents their concerns and their wish to notify supporting professionals. That way, reporting might feel less like “snitching” to bystanders, and more as a sign that they take the welfare of the family seriously.

Professionals have the know-how to help these families, but often feel that the problems are concealed from them. People surrounding these families often know that these families are struggling, but do not know what to do. One solution is to motivate bystanders to report signs of distress that they notice, another—additional—solution is to educate bystanders on what they can do to enhance parental motivation to seek care.

Our findings show that a more promising approach might be to stimulate bystanders to take responsibility to break the isolation of these families by showing a genuine interest in the underlying causes of their distress.

### Limitations of the Study

Since this was an explorative, grounded theory type of study, we only studied the role of bystanders indirectly: through the reports of parents and child welfare and addiction professionals. More research is needed on the experiences and contemplations of other bystanders., like professionals working at institutions parents and children attend for other reasons such as schools or child care centres and non-professional bystanders, like neighbours, friends or other parents at the playground.

Another characteristic of our study is that the data was collected from respondents who were already in treatment rather than those who are still reluctant or actively concealing their drinking behaviour. Some had already completed an intensive, in-patient family treatment of 9 months or had been in detoxification. Most respondents had already strongly benefited from care. So the results might not be representative for alcohol dependent parents who are still denying their alcohol dependency and are not in care. At the same time, it must also be said that the characteristic care-avoidance of (most) alcohol dependent parents would make it difficult to identify and recruit such parents for research purposes. However, most of the participating parents went through that phase as well, so they do remember their previous feelings and views well. The present article still provides important insights in how parents would have liked to be approached and guided toward sources of support and what had made them reluctant or defensive.

Our empirical data is collected among alcohol dependent parents, but not among parents with other drug-addictions (although some respondents had experience with other substances in the past). Whether our findings would also hold for them or for parents with multi-drug usage cannot be answered.

## Conclusion

Our interviews revealed that people (GPs, teachers, neighbours, family, friends) surrounding families in which parents struggle with problematic alcohol use are often aware of their distress, but are reluctant to intervene. This treatment gap is already well-known. The same counts for the insight that a non-judgmental approach toward parents with substance dependency can help them seek support. However, our study shows that, regardless of this state of knowledge, parents still experience stigma, and there is still a treatment gap—both in the eyes of parents as professionals.

By linking this treatment gap to the bystander effect, we hope to give new impulse to bridging the gap. One insight from general studies on the bystander effect is that it can be overcome when people know how to act.

We have provided some strategies on how to counteract the inertia and uncertainty caused by the bystander effect, guided by what distressed parents articulated as needing at the time of the intervention to open up and seek help. From the perspective of parents, effective access to care encouraged by empathetic professionals and other bystanders enquiring about “personal distress” and showing an interest in the parents as persons (not alcoholics) whilst encouraging them to seek help. However, this concern would be counterproductive if the bystanders focused mainly on any overt problems with alcohol misuse. Parents would feel that they were not being taken seriously, particularly if they regard their alcohol misuse as a symptom of underlying life stresses rather than as the actual cause of any troubles they have in leading their lives and parenting their children. Moreover, a focus on drinking problems would make them feel to be judged almost by definition as incompetent parents and may fear losing their children in child-custody cases. As a consequence, they might indeed avoid care altogether and not even dare to engage in any professional intervention. A non-judgmental approach that shows direct and explicit interest in any underlying troubles of the parent and that offers concrete help for these issues might, in the end, be the most encouraging and effective way for parents to also seek support for their alcohol dependency. However, the causes of their alcohol dependency should be acknowledged first, before the symptoms can be dealt with.

Of course, there are particularly serious or acute cases which require immediate action to ensure child safety and well-being. The aim of this article, however, is to contribute to the prevention of these situations by adequately responding to signs of parental distress by bystanders. An approach focussing on the underlying problems might not only contribute to motivating parents to seek treatment, but might also contribute to sustainable changes in these families.

One cause of the bystander effect is that people do not act because they feel they do not know how they should act. Research shows that many people feel uncomfortable with (mandatory) reporting systems (Thompson-Cooper et al., 1993; Greipp, 1997; Kalichman, 1999; Goldsberry, 2001). These bystanders fear that the vulnerable families they want to help would feel scrutinised and disciplined rather than supported. Our research shows a different way in which bystanders can react to signs of parental distress and we point toward possibilities to use these in public health campaigns.

## Data Availability Statement

The datasets presented in this article are not readily available because restrictions from the ethics committee. Requests to access the datasets should be directed to Anke Snoek (a.snoek@maastrichtuniversity.nl).

## Ethics Statement

The studies involving human participants were reviewed and approved by Research Ethical Committee of the Academic Hospital Maastricht and Maastricht University, positive advice has been granted (no 164216). The patients/participants provided their written informed consent to participate in this study.

## Author Contributions

AS designed and set-up the study together with DH. AS conducted the interviews, made a first analysis, and drafted the manuscript. DH conducted an independent analysis of the data and substantially contributed to revisions of the manuscript. WM and MV helped with the recruitment of respondents. BD, WM, MV, and GD all extensively commented on previous versions and significantly contributed to the final version of the manuscript. All authors agree with the final version.

## Conflict of Interest

The authors declare that the research was conducted in the absence of any commercial or financial relationships that could be construed as a potential conflict of interest.
